# Effects of Graphene Nanoplatelets on Mechanical and Fire Performance of Flax Polypropylene Composites with Intumescent Flame Retardant

**DOI:** 10.3390/molecules26134094

**Published:** 2021-07-05

**Authors:** Imran Ali, Nam Kyeun Kim, Debes Bhattacharyya

**Affiliations:** Centre for Advanced Composite Materials, Faculty of Engineering, The University of Auckland, 314 Khyber Pass Road, Newmarket, Auckland 1142, New Zealand; nam.kim@auckland.ac.nz (N.K.K.); d.bhattacharyya@auckland.ac.nz (D.B.)

**Keywords:** natural fibers, fire retardant, nano composite, encapsulation, mechanical properties, computational fluid dynamics

## Abstract

The integration of intumescent flame-retardant (IFR) additives in natural fiber-based polymer composites enhances the fire-retardant properties, but it generally has a detrimental effect on the mechanical properties, such as tensile and flexural strengths. In this work, the feasibility of graphene as a reinforcement additive and as an effective synergist for IFR-based flax-polypropylene (PP) composites was investigated. Noticeable improvements in tensile and flexural properties were achieved with the addition of graphene nanoplatelets (GNP) in the composites. Furthermore, better char-forming ability of GNP in combination with IFR was observed, suppressing HRR curves and thus, lowering the total heat release (THR). Thermogravimetric analysis (TGA) detected a reduction in the decomposition rate due to strong interfacial bonding between GNP and PP, whereas the maximum decomposition rate was observed to occur at a higher temperature. The saturation point for the IFR additive along with GNP has also been highlighted in this study. A safe and effective method of graphene encapsulation within PP using the fume-hood set-up was achieved. Finally, the effect of flame retardant on the flax–PP composite has been simulated using Fire Dynamics Simulator.

## 1. Introduction

Cellulose-based natural fibers, owing to their biodegradability, high specific strength and cost-effectiveness are being increasingly used in composites [1]. Polypropylene (PP), due to its easy processability, recyclability and good mechanical properties, such as tensile and flexural strengths, is widely utilized as the matrix for natural fiber-reinforced composites [2]. However, both natural fibers and polypropylene lack fire retardancy, and their composites are highly flammable. Under the influence of fire, natural fiber-reinforced composites start decomposing around 300 °C, releasing combustible gases, liquids, char and smoke, followed by rapid dripping. This can be hazardous and can lead to other ignition sources. Therefore, there is a need to improve the flame retardancy of natural fiber-based thermoplastic composites for engineering applications [3].

PP, due to its aliphatic hydrocarbon structure, burns rapidly with a nearly smoke-free flame and zero char residue. The pyrolysis process in polypropylene is initially due to random chain scission, generally at the carbon–carbon bond adjacent to the labile tertiary hydrogen atom in the repeat group. The polymer, when heated within the temperature range of 320 to 400 °C, starts decomposing and generates a mixture of clean hydrocarbon fuels together with lubricants. As the temperature exceeds 400 °C, isotactic PP releases maximum volatile products, which are comprised of dienes, alkanes and alkenes. The polymer ignites on reaching a temperature of around 570 °C. The high flammability of the polymer is mainly due to its fuel-forming tendency under heating [4,5].

Thermal degradation for lignocellulosic fibers usually start around 180 °C, which means matrices with low processing temperatures, such as polypropylene, polyethylene, etc., are suitable to manufacture composites. [1]. The plant fiber comprises cellulose, hemicellulose and lignin along with traces of water-soluble compounds, waxes and non-flammable substances referred to as ash [6,7]. Higher cellulose content results in higher flammability of fiber, whereas higher lignin content leads to char formation [8]. The addition of lignocellulosic fiber to polymer enhances the mechanical properties, such as tensile and flexural strengths; however, high flammability of such composites limit their applications in certain areas like transportation and aerospace [7,9].

The incorporation of flame retardants (FRs) into the thermoplastic polymer composites significantly reduces their flammability [10,11,12,13]. In recent years, halogenated FRs have been restricted due to concerns over the release of toxic gases and smoke during the burning process. At the same time, mineral-based FRs are not considered to be that effective due to their high loading requirements and detrimental effects on the mechanical properties. This has led to the widespread research on developing a more effective and environmentally friendly FR system. Intumescent Flame Retardant (IFR) systems are gaining popularity as they are halogen-free, eco-friendly and relatively efficient compared to other commercial FRs [14,15]. An IFR system consists of mainly three elements, namely an acid source, a carbonic source and a blowing agent. Synergistic interaction between these elements forms a swollen multicellular char, which can protect the underlying polymeric material from radiant heat flux or flame. The intumescent char can cease the polymer combustion by reducing the heat transfer and diffusion of volatile products between the flame and underlying material. The absence of dioxin and halogen acids also contributes towards reducing the toxicity during combustion [16,17]. IFR, such as ammonium polyphosphate (APP), utilizes phosphorus–nitrogen synergism by producing nitrogen and ammonia, which results in the dilution of fuel gases during combustion. At elevated temperatures, the IFR decomposes to form intumescent char, which acts as a barrier to limit the heat, fuel and oxygen transfer between the heat source and the underlying polymer, thus preventing the spread of fire [18,19].

**Figure 1:**
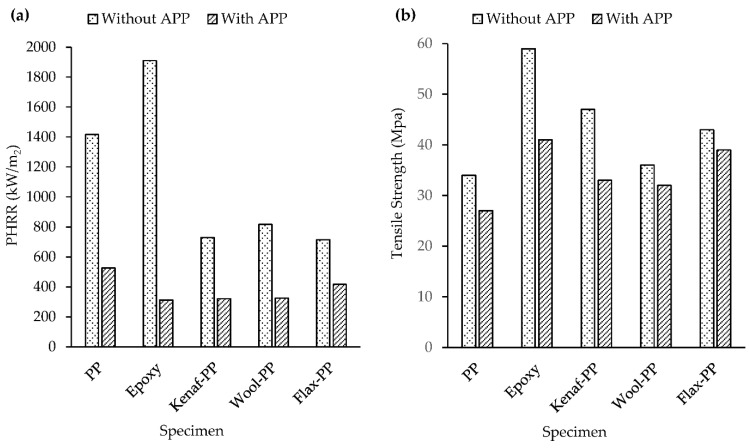
Effects of APP on (**a**) Peak heat release rate and (**b**) tensile strength of polymers and their composites.

Subasinghe et al. [10] studied the effect of three different intumescent APP-based flame retardants on the flammability and degradation of kenaf/PP composites. The authors observed through morphological analysis that compounding with a twin-screw extruder resulted in an effective dispersion of kenaf fiber and APP particles within the matrix. Significant drops in PHRR and THR, as compared to those of neat PP, were reported when 20 wt% APP was added to the composite. However, mechanical tests revealed reductions in tensile and flexural strengths of the composite. When kenaf was replaced by another natural fiber, such as flax, APP showed similar results, with reductions in PHRR and THR by 42 and 10%, respectively [11]. Figure 1a,b presents a comparison between PHRR values and tensile strengths of various polymers and their composites, with and without APP data collated from [10,11,20,21,22]. Based on the aforementioned studies, it is worthwhile to note that although intumescent flame retardants significantly improve the flame retardancy, they have a detrimental effect on the mechanical properties of the composites. The incorporation of IFR particles into a composite hinders the fiber/matrix interfacial bonding, and is responsible for the initiation of microcracks leading to the failure of the composite [14].

Recently, polymer nanocomposites have drawn considerable attention due to their excellent mechanical, electrical and thermal properties upon addition of a small quantity of nanofiller to a polymer matrix. Graphene, in its single-layer form, is found to be stronger than steel (Young’s modulus around 1TPa) and has a thermal conductivity (5000 W/m^−1^K^−1^, at room temperature) which is more than twice than that of diamond. Unlike micro- and macro-scale additives, graphene as a nanofiller has large surface area to volume ratio, which makes it possible to alter the mechanical strength of the matrix, by acting as a strong reinforcement. However, good dispersion of graphene nanoplatelets (GNP) with minimum agglomeration is vital [23,24]. Processing techniques for graphene-based polymer composites, such as solution mixing, melt blending and in-situ polymerization, play a crucial role in enhancing the dispersity of graphene particles within the matrix [25,26,27]. Achaby et al. [28] studied the mechanical performance of graphene-polypropylene nanocomposites. The authors reported a substantial increase in tensile strength (100%) and Young’s modulus (81%) at 3 wt% of graphene nanoplatelets as compared to those of neat polypropylene. Rafiee et al. [29] reported the enhancement of tensile strength and Young’s modulus by 40% and 31%, respectively, by incorporating just 0.1% weight fraction of graphene.

This research aims at investigating the effects of graphene nanoplatelets on the mechanical and flammability characteristics of flax polypropylene composites with intumescent fire retardant, through a set of comprehensive experiments. In this material system, APP acts as an intumescent flame retardant, short flax fiber and graphene platelets act as reinforcements, MAPP (Maleic Anhydride Polypropylene) acts as a binding agent and polypropylene is the matrix. It is anticipated that the incorporation of GNP into the composite will compensate for the loss in mechanical properties caused by FR additives. To the best of our knowledge, this is the first study to emphasize the mechanical performance of intumescent flame-retardant-based flax polypropylene nanocomposites. Furthermore, a numerical model using the Fire Dynamics Simulator (FDS) has been developed to predict fire parameters, such as heat release rate and time to ignition, for the flax–PP composite with IFR.

## 2. Results and Discussion

### 2.1. Mechanical Properties

**Figure 2:**
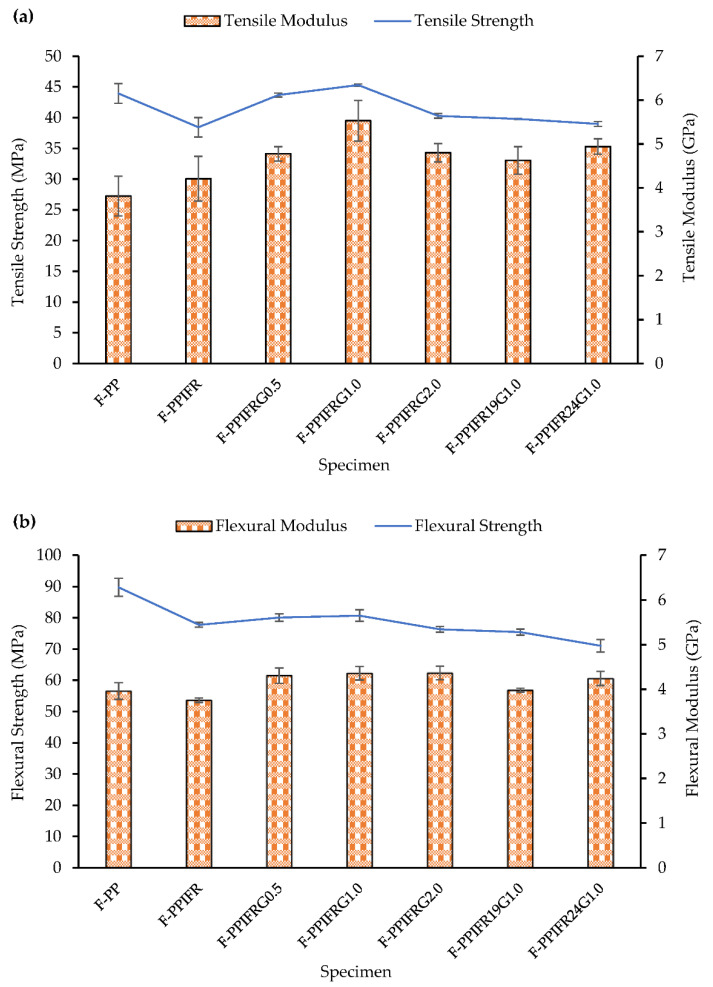
(**a**) Tensile and (**b**) flexural properties of composites.

The addition of 15 wt% IFR to the flax fiber-reinforced PP composite results in reduced tensile strength by 12.5%, compared to the F-PP composite, as shown in Figure 2. It has been reported that the physical presence of IFR hinders the interfacial adhesion between fiber and polymer, which leads to the formation of microcracks, resulting in failure of the composite [11,14,30]. However, the same IFR incorporated composite shows an increase of 10.3% in tensile modulus, which can be attributed to high stiffness and particulate nature of IFR additives [11,30,31].

An improvement in tensile strength by 13.6% was achieved when graphene nanoplatelets (0.5 wt%) was added to the composite (F-PPIFRG0.5). A further increase in tensile strength by 17.8% is observed in F-PPIFRG1.0, as compared to F-PPIFR composite. This is 3% higher than that of F-PP composite. The enhancement in tensile strength of the composite can be attributed to GNP’s intrinsic mechanical characteristics and its strong interfacial bonding with PP, resulting in higher interfacial stress transfer efficiency [28,32,33]. However, at 2.0 wt% GNP loading (F-PPIFRG2.0), the composite’s tensile strength is reduced by 11%, which is possibly due to the poor dispersion of GNP particles within the PP matrix, leading to GNP agglomeration at the fiber–matrix interface. A high amount of additives lessens fiber–matrix adhesion, consequently leading to reduced tensile strength [34]. 

A similar trend is observed for Young’s modulus, where 1.0 wt% GNP addition provides 45% enhanced tensile modulus as compared to that of the flax-reinforced PP composite. The high stiffness and aspect ratio of GNP contribute towards improved tensile moduli of the polymer composites [28,35]. Therefore, it can be concluded that incorporating 1.0 wt% GNP in the IFR composite offered significant improvement in mechanical properties. Moreover, composites with 19 and 24 wt% IFR and 1.0 wt% GNP exhibit comparable tensile strength and slightly higher tensile modulus than that of the F-PPIFR composite.

The flexural properties of the composites show a similar trend as the tensile properties, as shown in Figure 2b. The addition of IFR to the F-PP composite deteriorates the flexural strength by 13.5%, which is possibly due to weak interfacial bonding between IFR additive and polymer matrix. However, the presence of 0.5 and 1.0 wt% GNP only slightly improves the flexural strength by 3% compared to the F-PPIFR composite. A further reduction in flexural strength is observed in the F-PPIFRG2.0 composite. The flexural strength continues to drop with the increasing amount of IFR additive. All three composites with 0.5, 1.0 and 2.0 wt% GNP, display a similar flexural modulus, which is around 10% higher than the F-PP composite and 16% higher than the F-PPIFR composite.

### 2.2. Morphological Analysis

**Figure 3:**
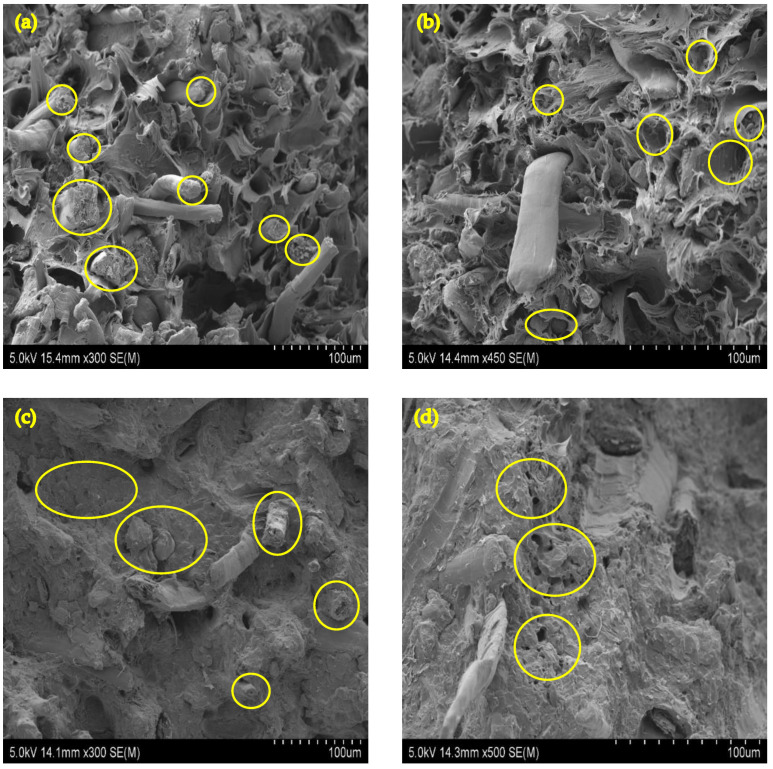
SEM images of tensile fractured surface of various types of composites: (**a**) F-PP, (**b**) F-PPIFR, (**c**) F-PPIFRG1.0 and (**d**) F-PPIFRG2.0.

Fractured cross-sections of composites from the tensile test were observed under SEM. Figure 3a shows that the F-PP composite has more fractured fibres’ surfaces than fibres pulled out, suggesting good interfacial bonding between flax fibres and PP matrix. The effect of IFR on fibre matrix interface is detected in Figure 3b. The presence of APP particles on the interface between fibre and polymer matrix (yellow circles) hinders the interfacial adhesion, leading to an increase in void content and fibres pulled out without breaking under tension. This results in reduced tensile strength in comparison with those of the composite without IFR. Reasonably uniform dispersion of 1.0 wt% GNP in the polypropylene matrix with low void content can be observed in Figure 3c. This indicates a good interaction between the GNP and PP matrix. Moreover, a large number of fractured surfaces of fibres can be observed in spite of the existence of IFR particles. On the other hand, Figure 3d shows poor dispersion of 2.0 wt% GNP in the composite, leading to graphene agglomeration and a large number of voids, thereby reducing the composite’s tensile strength. One possible reason could be an insufficient amount of polymer matrix to interact with GNP. These morphological analyses can support that the adverse effects of IFR particles on the fibre matrix bonding are diminished by the GNP and PP matrix surrounding them.

### 2.3. Cone Calorimeter Analysis

**Figure 4:**
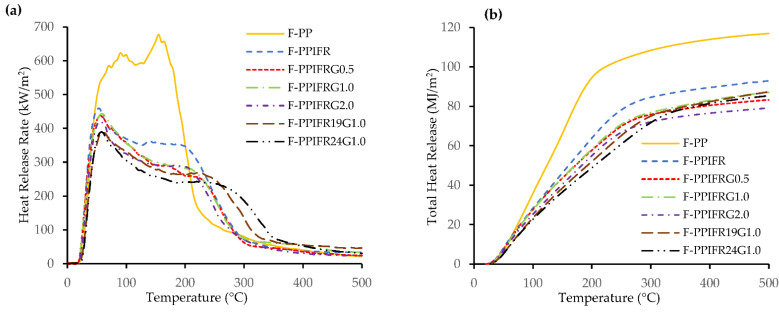
(**a**) Heat release rate and (**b**) total heat release of composites.

**Figure 5:**
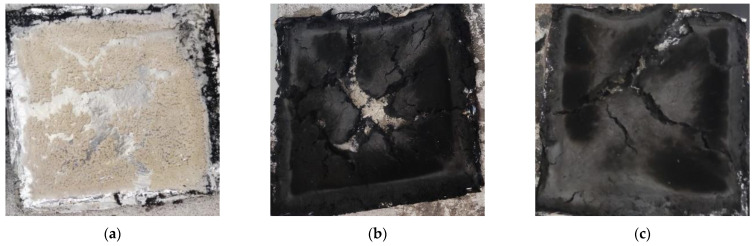
Char residue of (**a**) F-PP, (**b**) F-PPIFR and (**c**) F-PPIFRG1.0.

The addition of IFR significantly reduces PHRR and THR of the F-PP composite, as seen in Figure 4a,b. The intumescent char layer traps the flammable volatile products of PP and flax, thus cutting off the oxygen supply and suppressing the heat release rate curve [36]. Figure 5a shows that the incorporation of GNP further reduces the HRR curve compared to the F-PPIFR composite. It can be inferred that GNP can contribute to the formation of the compact char structures. Yuan et al. have reported a synergistic effect of graphene and IFR on the intumescent char formation to enhance the flame-retardant performance of PP composites [37]. Figure 5a–c illustrates the char residue of F-PP, F-PPIFR and F-PPIFRG1.0 composites post cone calorimeter analysis. The char residue from F-PPIFRG1.0 shows denser structure with relatively less cracks compared to F-PPIFR, whereas no char layer is formed for the F-PP composite. In addition, the increase in IFR content from 15 to 19% leads to the PHRR reduction by 11.6%, but the composite with IFR content more than 19 wt% does not show any significant improvement. It can be suggested that the amount of IFR additive reached a saturation point beyond which there is no noticeable effect in the fire performance of the composite. 

Table 1 presents the fire performance index (FPI) of each composite as a ratio of time to ignition (TTI) to PHRR. The FPI value can indicate the size of fire hazard and higher FPI value refers to lower fire hazard [10]. Among all the composites tested, the flax–PP composite with 19 wt% IFR and 1.0 wt% GNP demonstrates the highest FPI value. At a later stage, composites with IFR produced a higher amount of CO ((kg/kg) is the fraction of fuel mass converted into carbon monoxide) than the composites without IFR mainly because the char layer formation leads to incomplete combustion, which suppresses the oxidation process during the test, thus increasing CO and lessening CO_2_ production compared to the composite without IFR [30].

### 2.4. Thermogravimetric Analysis

**Figure 6:**
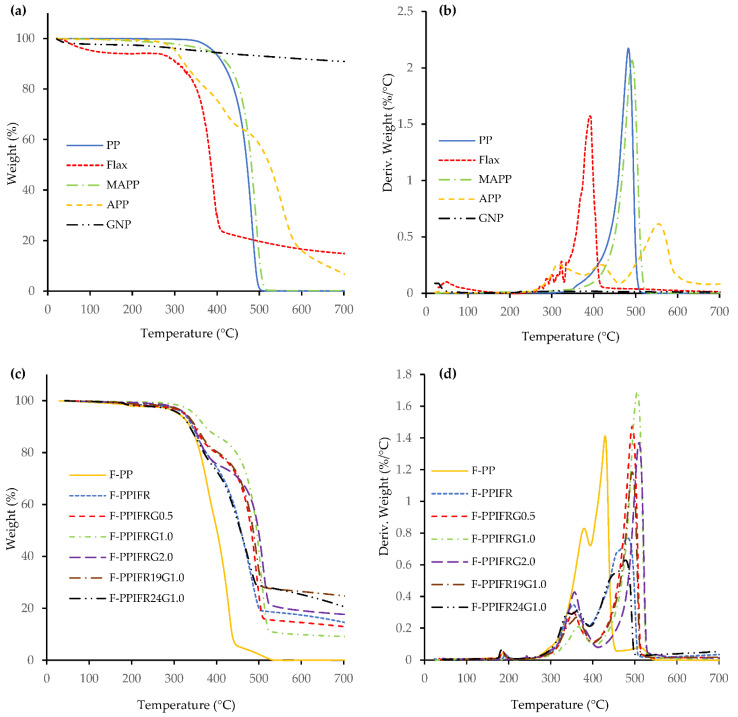
TG and DTG curves of neat materials (**a**,**b**) and their composites (**c**,**d**).

Flax fiber starts losing its weight at 100 °C because of moisture evaporation, as seen in Figure 6a. It begins to decompose at around 270 °C, whereas the maximum decomposition rate is observed at about 390 °C. The degradation between 270 and 370 °C is primarily due to the decomposition of hemicellulose, releasing a large amount of methane. The final stage of decomposition between 370 and 550 °C corresponds to the degradation of α-cellulose due to depolymerization. Lignin content in flax, responsible for char yield, decomposes in the temperature range of 200–550 °C [38]. Furthermore, GNP shows the negligible weight loss at elevated temperatures, as seen in Figure 6a. APP is observed to have two decomposition stages at 325 and 550 °C. The first stage is due to the removal of volatiles such as NH_3_ and H_2_O and the formation of crosslinked polyphosphoric acids, whereas the second stage is attributed to the evaporation of polyphosphoric acids and dehydration to phosphorous pentoxide (P_4_O_10_). The TGA and DTG curves for composites illustrate two stages of thermal degradation process, and T_max1_ and T_max2_ denote the corresponding temperatures in Table 2. The char residue of flax–PP composite significantly increases from 0.2 to 18 wt% when 15 wt% IFR is added to the composite. Furthermore, a substantial drop in the decomposition rate (T_max2_) is observed, as can be seen in Figure 6d.

Interestingly, the F-PPIFR1.0 composite shows the lowest decomposition rate at T_max1_ (around 370 °C) but the highest decomposition rate at T_max2_ (about 500 °C). It is understood that strong interfacial bonding between PP and GNP resulted in higher resistance against thermal degradation at T_max1_, but as the temperature increases, the interfacial adhesion begins to weaken, resulting in a higher decomposition rate [11,39]. F-PPIFRG2.0, due to poor interfacial adhesion between PP and GNP, displays a higher decomposition rate at T_max1_, whereas the second stage of decomposition occurs at a higher temperature because of the high thermal resistivity of GNP. The addition of 19 and 24 wt% IFR to the F-PP composite with 1.0 wt% GNP leads to further reduction in the decomposition rate, with the increase in final residue at 700 °C [22].

### 2.5. Pyrolysis Model

**Figure 7:**
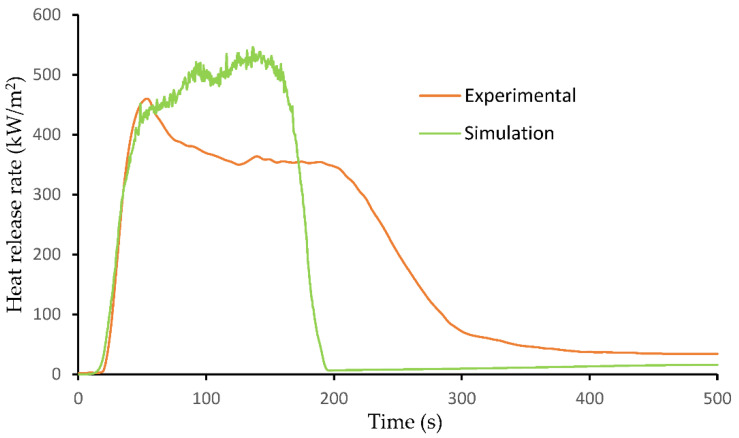
Experimental and numerical HRR curves of IFR-based flax–PP composite at 50 kW/m^2^.

Figure 7 illustrates the experimental and simulated HRR curves of the IFR-based flax PP composite (F-PPIFR). The predicted HRR curve reasonably matches the experimental result at the early stage due to the well-pyrolysis model using proper material properties in FDS. In addition, compared to a previous study done by Kim et al. [40], where they simulated a flax–PP composite using FDS, the flame-retardant effect of APP can be observed in the present study, with a reduction of PHRR from 700 to 530 kW/m^2^. However, the difference in PHRR between simulated and experimental results is identified since simulation of the char-forming mechanism is limited in the software package. The reaction order of 1.0, which is the default value in FDS, was applied to this model. The reaction order of a pyrolysis reaction determines the dependence of the reaction rate on the concentration of species [41,42]. The burning process in the model ends at around 190 s, which might be due to a higher reaction rate resulting in faster burning of fuel, which is the composite polymer and flax fibers. Therefore, possible future work to improve the accuracy includes a char-forming model and reaction order calculation.

## 3. Materials and Methods

### 3.1. Materials

Flax fibers were supplied by Bruce Smith Ltd. (Auckland, New Zealand). The polymer matrix was Moplen HP-400N polypropylene (Lyondell Basell, Auckland, New Zealand) with a melt flow index of 11 g/10 min (based on ISO 1133-1 standard under 190 °C and 2.16 kg). Maleic anhydride grafted polypropylene (MAPP) (Licocene 6452, Clariant, New Zealand) with 7 wt% maleic anhydride was used as a compatibilizer. Ammonium Polyphosphate (APP) (Budit 3167, Budenheim, Germany) was selected as the FR in this study. This commercially available halogen-free intumescent flame retardant contained 22 wt% of phosphorous and 21 wt% of nitrogen, respectively. Graphene powder with carbon content >92% (Changsha Easchem Co., Ltd., Changsha, China) was used as the nanofiller. The average particle size was 4–8 nanometer with a thickness of 1–3 layers.

### 3.2. Graphene Encapsulation

Due to safety reasons, graphene nanoplatelets were melt mixed with polypropylene inside a fume hood (Air Science PurAir Basic Ductless), equipped with HEPA (High-Efficiency Particulate Air) filters to prevent the spreading of GNP into the atmosphere. GNP was mixed with pulverized PP in a Teflon-coated mold placed inside the fume-hood. Pulverized PP was used for this process because of the high surface area. GNP/PP mixture had a ratio of 1:20 by weight. First, 2.5 g of GNP was mixed with 50 g to prepare one of the several batches. Any ratio higher than 1:20 resulted in improper mixing of GNP with PP. After mixing GNP/PP in their powdered form, the mold was heated to 200 °C, and the mixture was stirred manually for 2–3 min until no graphene particles were visible at the surface. The mold was then quenched to around 50 °C by subjecting it to cold water to obtain GNP/PP sheets. These sheets were then flattened using a hot press at 160 °C and again granulated.

### 3.3. Composite Manufacturing

In this study, short fibers (average length 2.9 mm) were prepared by a granulator (GRV variant series, Italy), with a 5 mm grid mesh plate, and then dried in a vacuum drier at 80 °C for 60 h. Flax fibers, neat PP, GNP/PP powder and other additives, such as IFR and MAPP, were dry-mixed using Phas-o-mec high-intensity mixer at 18 Hz for 7 minutes. The constituent’s dry mixture was directly processed in the LTE 26–40 Lab Tech twin-screw extruder for melt blending. The screw (length L/diameter D ratio of 40) rotated at 120 rpm under an average processing temperature of 175 °C to attain efficient mixing of fibers and other additives within the molten PP. The Boy 50A injection molding machine was employed to manufacture mechanical test specimens out of compound pellets. The temperature profile from the feeder to the nozzle was 170/175/175/180 °C, and the injection pressure was about 85 bar. The dimensions of tensile and flexural test specimen were based on ASTM D638 (50 mm gauge length) and ASTM D790 (3.2 mm × 12.7 mm × 125 mm) standards. The cone calorimeter test samples (100 mm × 100 mm × 3 mm) were prepared through compression molding using the Scientific Lab Tech Compression Molding machine. The compound pellets were subjected to temperature, pressing load and pressing time of 220 °C, 50 bar and 60 s, respectively. Table 3 shows the composition of composites selected for this study. Since flax fibers and PP have different fire and mechanical behavior, the idea behind choosing these compositions was to maintain a constant weight percentage ratio among the three fundamental constituents (flax, PP and MAPP), which are typical for all the composites. The aim was to study the effect of APP and GNP on flax/PP/MAPP composite. Once the optimum wt% of GNP to improve the mechanical properties was determined, samples with a higher amount of IFR (19 and 24%) were manufactured, while keeping GNP wt% constant, to further improve the fire properties.

### 3.4. Characterisation

Thermal decomposition of flax fiber, neat polypropylene, graphene nanoplatelets, ammonium polyphosphate and composites was studied using thermogravimetric analysis instrument (TGA-50, Shimadzu, Japan). Approximately 10 mg of the specimen was heated from ambient temperature (~25 °C) to 700 °C at the rate of 10 °C/min in an inert (N_2_) atmosphere. The morphological analysis of fractured cross-sections of composites was carried out using scanning electron microscope (Hitachi SU-70). The specimens were coated with platinum using Quorum Q150RS sputter coater (Quorum Technologies Ltd., East Sussex, UK) to enhance the conductivity of the surface.

### 3.5. Cone Calorimeter

Combustion properties of composites were analyzed using a cone calorimeter (FTT Limited, East Grinstead, UK) based on ASTM E1354 international standard. The compression molded specimens were pre-conditioned at 23 °C and 50% relative humidity for 48 h, before being tested under an external heat flux of 50 kW/m^2^. The cone calorimeter installed with Servomex 1440 oxygen analyzer (Servomex, UK) can measure a large range of quantifiable data of the combustion parameters, such as heat release rate (HRR), mass loss rate, smoke production rate, etc. For this paper, the focus was mainly on the HRR as it is considered to be the most important fire reaction property to measure thermal energy, responsible for the growth and spread of fire [41,42,43,44]. Three samples for each composite were tested to get an average value.

### 3.6. Mechanical Testing

Tensile strength and modulus (chord modulus between 0.05 and 0.25% strain) of the composites were measured using Instron 5567 Universal Testing Machine (UTM), based on the ASTM D638 standard. Furthermore, flexural strength and modulus were obtained under a 3-point bending rig based on ASTM D790 standard. Five tests for each composite (total of seven types of composites) were carried out to get the average mechanical properties.

### 3.7. Numerical Modeling

A computational fluid dynamics (CFD) model was developed in Fire Dynamics Simulator (FDS), to simulate heat release rate of F-PPIFR composite. FDS computes a form of the Navier–Stokes equations, which is suitable for low-speed, fire-driven fluid flow, where the phrase low speed refers to gas velocities lower than a Mach number of 0.3 [45]. The turbulence model is developed by employing the standard Smagorinsky form of Large Eddy Simulation (LES). The term LES can be described as turbulent mixing of the gaseous fuel and combustion products. The low Mach number equations are solved numerically by dividing the physical space where the fire is to be stimulated into rectangular cells. FDS includes an algorithm based on the second-order Runge–Kutta predictor-corrector scheme for updating velocity, pressure, temperature and other essential variables in time [44,45,46,47,48].

Physical and thermal properties of the composite’s constituents such as flax, PP and APP as well as char residue were the input parameters in the FDS code and are shown in Table 4. Furthermore, in the gas phase ‘mixing controlled’ combustion model, chemical formulae for flax and PP were defined as C_6_H_10_O_5_ and C_3_H_6_, respectively, and molecular weight of APP was ~97.01 g/mol. All these gas-phase reaction fuels were considered as lumped species to reduce the number of transport equations, thus reducing the computational power. The solid-phase pyrolysis process is based on a single step global Arrhenius reaction [49]. Thermogravimetric analysis (TGA) was conducted on the composite constituents to get the pyrolysis reaction input parameters, such as reference temperature, heating rate and pyrolysis range. TGA results determined char yield of each reaction fuel and the IFR-based composite. Since FDS is limited to simulating char formation of the composites with flame retardants, we defined the char as a material and added its thermal and physical properties in the code. The following assumptions were made to develop the FDS model:

1. Flax and PP/APP char residues were assumed to have the same thermophysical properties.
2. Based on the TGA result, the char residue of F-PPIFR composite was measured to be 18 wt%. Therefore, PP was assumed to form char as a by-product due to reaction with APP in the numerical model, as shown in Table 5.

The computational domain of 400 × 400 × 500 mm was defined according to the cone calorimeter’s physical configuration based on ASTME E-1354 standard. The free flow of air was allowed to enter the domain by setting the bottom surface to ‘open boundary’ conditions. A duct with a volume flow rate of 24 L/s was set up on the domain’s top surface to imitate the cone calorimeter exhaust system. The heat flux of 50 kW/m^2^ was established by setting the cone heater temperature to 750 °C. The cell size of 0.67 mm was assigned in this numerical model to compute the heat release rate.

## 4. Conclusions

This study provides insight into the effects of graphene nanoplatelets on the mechanical and fire properties of the intumescent flame-retardant-based flax polypropylene composites. Tensile and flexural properties of flame-retardant composites with and without GNP were investigated. Strong interfacial bonding between GNP and PP was identified when 1.0 wt% GNP was added to the FR composite, with an increase of 17.8% and 31.4% in tensile strength and modulus, respectively. However, the addition of 2.0 wt% GNP in the FR composite improved the tensile strength by just 4.5%, due to GNP agglomeration. The graphene nanoplatelets also contributed to forming a relatively denser and rigid char, resulting in the reduction of PHRR and THR by 8.5 and 12.3%, respectively. The cone calorimeter tests showed the suppression of HRR curves after the initial peak in composites incorporated with GNP compared to the flame-retardant composite without GNP. When the IFR loading was increased to 19 wt%, PHRR was further reduced by 15%; however no significant improvement in fire behavior was observed with 24 wt% APP, suggesting that IFR saturation had reached beyond 19% by weight. Furthermore, the lowest decomposition rate was observed at T_max1_ in composite with 1.0 wt% GNP due to strong interfacial bonding between GNP and PP. The addition of GNP also increased T_max2,_ which infers that the maximum decomposition rate occurred at higher temperatures for FR nanocomposites in comparison with flame-retardant composites. A safe way of encapsulation of graphene nanoplatelets was achieved in this study using fume-hood. For future work, techniques like in situ polymerization can be employed for better mixing of graphene nanoplatelets within the polypropylene. Furthermore, the CFD model developed in FDS could simulate a reasonable effect of flame retardancy on the IFR-based flax–PP composite, particularly during the onset of the burning process. However, deeper investigation is required with regards to pyrolysis reaction rate, kinetic parameters and char-forming mechanism during the combustion process.

## Figures and Tables

**Table 1:** Cone calorimeter results.

Specimen				Cone Calorimeter Results			
	TTI (s)	PHRR (kW/m^2^)	TPHRR (s)	THR (MJ/m^2^)	FPI (m^2^s/kW)	CO (kg/kg) (±0.004)	CO_2_ (kg/kg) (±0.04)
F-PP	23.7 ± 1.5	677.7 ± 12	155 ± 1	118.2 ± 2.4	0.034	0.027	2.47
F-PPIFR	20 ± 2.6	459.5 ± 7	53.4 ± 2	89.9 ± 2.3	0.043	0.089	1.80
F-PPIFRG0.5	20 ± 2	439.1 ± 16	56.7 ± 2.8	84.16 ± 4.6	0.045	0.101	1.74
F-PPIFRG1.0	19.6 ± 1	442.8 ± 11	60 ± 0.5	88.6 ± 6	0.044	0.093	1.76
F-PPIFRG2.0	19.7 ± 0.5	420.5 ± 12	56.7 ± 2	78 ± 1.4	0.046	0.091	1.07
F-PPIFR19G1.0	23.2 ± 2	391.4 ± 1	60 ± 5	92.2 ± 3.1	0.065	0.106	1.58
F-PPIFR24G1.0	25 ± 0.5	388 ± 8	58.3 ± 2	86.8 ± 0.9	0.064	0.096	1.57

**Table 2:** TGA results of PP, Flax, APP and various composites.

Specimen	T_5%_ (°C)	T_max1_ (°C)	T_max2_ (°C)	Residue at 700 °C (wt%)
PP	391	483	-	0
Flax	109	393	-	15
APP	302	318	550	10
F-PP	341	395	-	0.2
F-PPIFR	337	360	477	18
F-PPIFRG0.5	330	360	497	18.5
F-PPIFRG1.0	350	347	510	10.2
F-PPIFRG2.0	330	360	515	19.5
F-PPIFR19G1.0	330	352	495	23.5
F-PPIFR24G1.0	316	345	475	21.6

**Table 3:** Composition of the composites.

Constituent Content (wt%)					
Specimen	Flax	PP	MAPP	APP	GNP
F-PP	29.41	67.05	3.53	0	0
F-PPIFR	25	57	3	15.0	0
F-PPIFRG0.5	24.87	56.71	2.98	14.92	0.5
F-PPIFRG1.0	24.75	56.43	2.97	14.85	1.0
F-PPIFRG2.0	24.50	55.86	2.94	14.70	2.0
F-PPIFR19G1.0	23.51	53.65	2.82	19.0	1.0
F-PPIFR24G1.0	22.05	50.30	2.64	24.0	1.0

**Table 4:** The input parameters for the numerical model [46,47,48].

Property	Unit	Materials PP	Flax	APP	Char Residue
Density	Kg/m^3^	900	1400	1900	550
Emissivity	-	0.97	0.85	0.94	0.93
Specific heat capacity	kJ/kg K	1.651	1.6	4	2
Thermal conductivity	W/m K	0.18	0.3	0.4	0.1
Heat of combustion	kJ/kg	4.9 × 10^4^	9300	-	-
Heat of reaction	kJ/kg	1987	1411	880	-
Reference temperature	°C	482	391	625	-
Heating rate	°C/min	20	20	20	-
Pyrolysis range	°C	155	145	400	-
Residue yield	kg/kg	0.2125	0.15	0.1	-
Number of reactions		1	1	1	1

**Table 5:** Formulation of char residue for FDS based on thermogravimetric analysis.

Material	Constituent	Char Residue	Composite Char Contribution
	(wt%)	(wt%)	(wt%)
Flax	25	15	3.75
APP	15	10	1.5
PP	60	21.25	12.75

## Data Availability

Not applicable.

## Sample Availability

Samples of the composites are available from the authors.
